# Mechanical and Thermal Behavior of Hemp-Reinforced Starch/Agar Biocomposites: Insights from Finite Element Simulation and Machine Learning Models

**DOI:** 10.3390/polym17070855

**Published:** 2025-03-23

**Authors:** Ehsan Fartash Naeimi, Kemal Çağatay Selvi, İbrahim İnanç, Nicoleta Ungureanu

**Affiliations:** 1 Department of Agricultural Machinery and Technologies Engineering, Faculty of Agriculture, Ondokuz Mayis University, 55139 Samsun, Türkiye; agri.ehsan@gmail.com; 2 Department of Metallurgical and Materials Engineering, Faculty of Engineering, Ondokuz Mayis University, 55139 Samsun, Türkiye; ibrahim.inanc@omu.edu.tr; 3 Department of Biotechnical Systems, Faculty of Biotechnical Systems Engineering, National University of Science and Technology Politehnica Bucharest, 060042 Bucharest, Romania

**Keywords:** industrial hemp, alkaline/peroxide pretreatment, natural reinforcing agents, biocomposite films, finite element method, machine learning

## Abstract

The increasing effects of plastic pollution have led to the study of eco-friendly and biodegradable alternatives. The present study concerns the production and characterization of biocomposite films from starch, agar, and alkaline/peroxide-treated hemp fiber powder. In total, nine films were fabricated with variable ratios of starch–agar (0, 0.5, and 0.75) and hemp fiber (0, 15, and 30 wt.%). The physical, mechanical, and thermal properties of these films were evaluated. Tensile tests demonstrated that adding 30% hemp fiber increased Young’s modulus, while 15% fiber decreased tensile strength. In the SAH group, adding 1.5 g of agar significantly improved tensile strength and Young’s modulus, especially in the SAH30 sample. Finite element simulations of tensile tests showed remarkable agreement with experimental data. Machine learning models (SVM and GP) were used to predict tensile strength, with the SVM model using the RBF kernel showing the highest accuracy (R^2^ = 0.938). Impact tests indicated that resistance was improved by agar, with the SAH group showing optimal stress distribution and energy absorption. Steady-state and transient thermal analyses showed that hemp fiber increased thermal resistance, and heat stress depended mainly on composition, especially agar and fiber. This research accentuates the potential of hemp fiber and agar to improve the properties of starch-based films and thereby opens routes toward sustainable material development.

## 1. Introduction

Escalating concerns regarding the detrimental impacts of plastic pollution on both animal and human health have fueled a growing impetus for the exploration of biodegradable alternatives. Beyond ensuring optimal biodegradability, there is a burgeoning interest in the development of materials from renewable resources to mitigate the environmental footprint of plastics [1,2]. Modern biocomposite films are predominantly produced from renewable polymers such as polysaccharides, proteins, and lipids. Polysaccharides like starch [3], agar [4], cellulose and its derivatives [5], chitin, and chitosan [6] have garnered attention as packaging materials due to their inherent biodegradability and desirable barrier properties. Nevertheless, inherent weaknesses in mechanical and antimicrobial attributes necessitate the integration of other biodegradable constituents to reinforce polymeric matrices and augment their performance.

The increasing demand for biodegradable, sustainable, and recyclable materials has propelled the utilization of natural fibers, such as hemp, as reinforcing agents in composite formulations [7,8,9]. Most hemp biomass is derived from the lignocellulosic stem of the plant, which has diverse applications [10]. Industrial hemp is specifically cultivated for high-quality fiber production, yet each component of the plant can serve as a raw material for a diverse array of end products [11,12,13]. Given that the majority of hemp fibers are obtained as agricultural by-products, they are cost-effective and abundantly available, which can significantly reduce the production costs of final products [10].

The combination of hemp fibers with thermoplastic polymers offers numerous advantages, including recyclability, low density, high sound absorption, low abrasion, improved energy recovery, high strength-to-weight ratio, and non-toxicity. These renewable materials have demonstrated the potential to substitute synthetic materials across a variety of applications, such as seat backs, doors, and under-body panels in the automotive industry [14]. Prior studies have predominantly employed waste hemp fibers as fillers within the polymeric matrix of non-biodegradable materials (e.g., polyethylene and polypropylene) or synthetic polymers such as PVA [3,14,15,16,17]. However, these approaches have faced criticism due to technical and financial limitations, as well as environmental considerations. Technical challenges in the composites mentioned include problems like poor adhesion between hemp fibers and the polymer matrix, issues in the manufacturing process, and less-than-ideal mechanical and thermal properties of the final product.

In this study, to surmount these constraints, a novel approach was adopted focusing on the utilization of entirely biodegradable matrices and natural reinforcing agents. Accordingly, alkaline/peroxide pretreatment was explored to enhance the interfacial adhesion of hemp fiber. Subsequently, pretreated hemp fibers were incorporated within a starch–agar polymer matrix to fabricate biocomposite films with optimized performance. The mechanical and thermal properties of the films were simulated and analyzed using Abaqus 2021 (Dassault Systemes) and the finite element method (FEM). Simulation, as a powerful tool, provides the means for analyzing and optimizing material properties prior to production, thus significantly diminishing research and development time and costs [18]. Furthermore, based on the tensile test data, tensile strength values were predicted using Support Vector Machine (SVM) and Gaussian Process (GP) methods under varying conditions. Machine learning methods, employing advanced algorithms, enable the extraction of latent patterns from experimental data, thus assisting researchers in accurately predicting material properties and optimizing their designs [19]. The composites developed in this study hold substantial potential for broad utilization across diverse industries, including packaging, automotive, plumbing, and cosmetics, as a sustainable and eco-friendly alternative in the future.

## 2. Materials and Methods

### 2.1. Preparation and Sourcing of Raw Materials

Hemp fiber residues, including shives and hurd, were obtained from the Hemp Research Institute (Samsun, Türkiye). Agar powder (commercially known as agar-agar) and glycerol were purchased from Sigma-Aldrich, Inc. (St. Louis, MO, USA). Corn starch was procured from the Piyale brand (Samsun, Türkiye).

For the pretreatment of hemp fibers, sodium hydroxide (NaOH) solution and 23% hydrogen peroxide (H_2_O_2_), supplied by KimyaLab (Istanbul, Türkiye), were used. All raw materials were prepared and stored under standardized conditions to ensure quality and reliability throughout the experimental process.

### 2.2. Preparation and Pretreatment of Hemp Fiber

**Figure 1:**
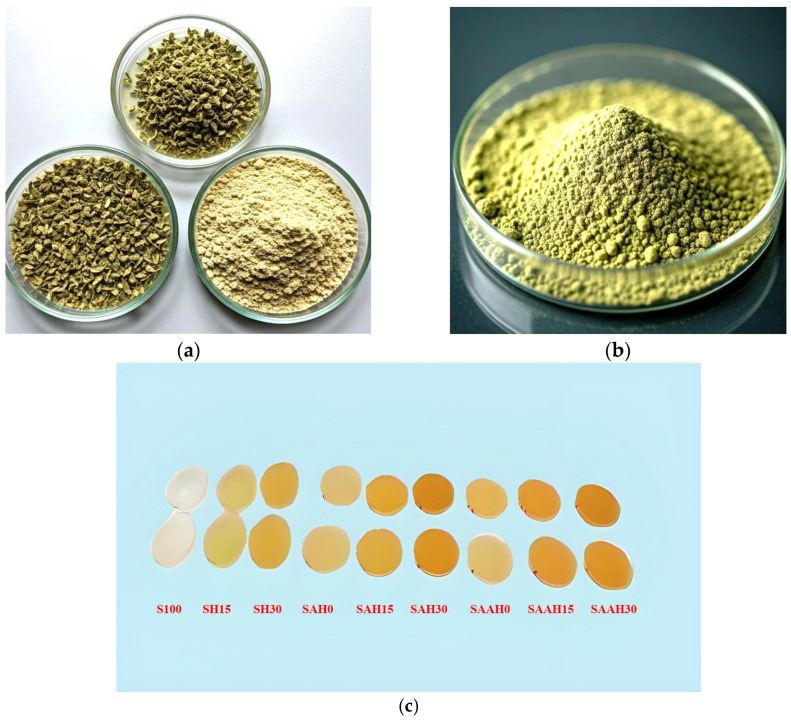
Conversion stages of hemp residues into: (**a**) 20-micrometer (635-mesh) powder; (**b**) alkaline/peroxide pretreated powder; (**c**) biocomposite films.

The shredded hemp fiber residues were dried at 90 °C for 24 h. The dried materials were ground into smaller fragments using a milling machine and sieved through a 635-mesh (20 μm) sieve to obtain a uniform powder (Figure 1a).

During the alkaline/peroxide pretreatment, 5 g of the hemp fiber powder was mixed with 300 mL of distilled water and subjected to ultrasonication for 30 min. The following step allowed for the dispersion of fibers and enhanced water penetration into their structure. After ultrasonication, the wet powder was filtered using a vacuum filter with a pore size of 20 μm to remove excess water. Afterwards, the filtered powder was treated with 100 mL of 8% sodium hydroxide (NaOH) solution (8% *w*/*v*) under ultrasonication for 10 min. This process effectively removed lignin and other non-cellulosic compounds from the fiber surface, improving its structural properties. To further improve the mechanical and chemical properties of the hemp fibers, a hydrogen peroxide (H_2_O_2_) treatment was carried out by following the protocol recommended by Momeni et al. (2021) [20]. To this end, 100 mL of 11% hydrogen peroxide solution (11% *w*/*v*) was added to the alkaline mixture, and the resulting solution was vigorously stirred at room temperature for 90 min to induce oxidation and surface modification of the fibers. The filtering step was carried out with a 20 μm vacuum filter, and extensive washing with distilled water was continued until the pH reached approximately 7. Finally, the obtained powder was completely dried in an oven at 90 °C for 24 h, making it ready for applications (Figure 1b).

### 2.3. Preparation of Biocomposite Films

Biodegradable films were prepared using the casting technique. Agar-to-starch ratios in film compositions were varied to 0, 0.5, and 0.75, while alkaline/peroxide-treated hemp fiber powders were maintained at 0, 15, and 30% (*w*/*w*) based on the total weight of starch and agar. The selection of starch–agar ratios and hemp fiber contents in this study was directed towards optimizing the compromise among the flexibility of films, mechanical strength, and biodegradability. By the systematic tuning of these parameters, the objective was to determine the optimal formulation that offers maximum mechanical and thermal performance along with environmental sustainability.

To prepare the base solution, 3.5 g of starch was dissolved with 2 mL of glycerol in 100 mL of distilled water. The solution was continuously stirred in a water bath at 90 °C for 15 min. Then, agar was added to the solution in the specified proportions and stirred again under the same conditions for another 15 min. In parallel, various formulations of alkaline/peroxide-treated hemp fiber powder (0, 15, and 30% *w*/*w*, respectively) were dispersed in 30 mL of distilled water with continuous stirring in a water bath at 90 °C for 15 min. The mixture was further sonicated for 15 min to complete the dispersion process of the fibers.

The hemp fiber dispersion was mixed at 75 °C under constant stirring for 15 min until it gained properties similar to a homogenous gel solution by agar/starch solution. The resulting mixture was cooled to 40 °C and stirred further to remove any air bubbles. Finally, 40 mL of the prepared solution was poured into Petri dishes and dried in an oven at 60 °C for 24 h (Figure 1c).

The film prepared from pure starch was considered the control sample and labeled S100. For the other films, the following abbreviations were used: S indicates starch, A indicates the incorporation of 1.5 g of agar, AA indicates the incorporation of 2.25 g of agar, and H indicates the incorporation of alkaline/peroxide-treated hemp fiber powder, while the numbers 0, 15, and 30 correspond to the percentage of hemp fiber powder added based on the total weight of starch and agar.

### 2.4. Evaluation of Samples

#### 2.4.1. Thickness and Density

The thickness of the films was measured with the aid of a precision digital micrometer. To ensure accuracy and representativeness, three random measurements were taken at different locations on each film sample, and the average values were recorded as the final thickness.

For density calculation, two reliable methods were employed: the Archimedes method and the volume–mass method. In the Archimedes method, the principles of buoyancy were applied to determine the density, while in the volume–mass method, the density was calculated by accurately measuring the volume and mass of the samples. Both methods were utilized simultaneously to ensure the accuracy and reliability of the results in evaluating the physical properties of the samples.

#### 2.4.2. Tensile Test and Finite Element Simulation

In this study, Young’s modulus, tensile strength, and elongation at break were determined using an Instron universal testing machine (Instron, Norwood, MA, USA) based on standard test methods [21]. Each film strip for testing was then carefully placed between the grips, keeping the distance between the grips at 5 cm. The crosshead speed was set to 10 mm/min to control the conditions during testing.

Tensile strength was calculated as the ratio of the maximum force required for film rupture to its initial cross-sectional area [22], in MPa. This test was carried out to measure, as precisely as possible, the resistance of the film to tensile stresses. Elongation at break was further determined by the calculation of the length of the film at a rupture point against its initial length and expressed in percentage form for material ductility during testing. The stress ($\sigma_{engineering}$) and strain ($\varepsilon_{engineering}$) data obtained from the testing machine were recorded in engineering units. These values were then converted into true stress ($\sigma_{true}$), true strain ($\varepsilon_{true}$), and plastic strain ($\varepsilon_{plastic}$) using Equations (1)–(3), respectively, to provide a more accurate representation of the behavior of the film under realistic conditions.

(1) $$\sigma_{true}=\sigma_{engineering}\cdot (1+\varepsilon_{engineering})$$

(2) $$\varepsilon_{true}=ln\;(1+\varepsilon_{engineering})$$

(3) $$\varepsilon_{plastic}=\varepsilon_{true}-\frac{\varepsilon_{true}}{E}$$

Finite element simulation was conducted to more accurately predict the behavior of the films under tensile testing conditions. The simulations enabled the modeling of mechanical responses, including the distribution of stress–strain in samples under different loading conditions. In the current study, Abaqus 2021 (Dassault Systemes) was employed to perform accurate mechanical and thermal simulations. First, a 3D model of the samples was created based on their real dimensions and conditions. Material properties, including thickness, density, Young’s modulus, tensile strength at break, and plastic strain (obtained during the tensile test or prior), were entered into the property section of the software. Boundary conditions and appropriate meshing were applied to the model. Finally, displacements along the y-axis for the upper grip and fixed conditions (x = y = z = 0) were given for the lower grip to simulate real testing conditions.

#### 2.4.3. Machine Learning Models

Support Vector Machine (SVM) and Gaussian Process (GP) methods were utilized as two machine learning techniques to predict the tensile strength of the composite films. The SVM model was selected due to its high capability in classifying and performing regression on non-linear data, while the GP model was used to generate probabilistic predictions and quantify uncertainty [23].

For each film, three repetitions were conducted, and data including thickness, density, yield stress, tensile strength and maximum stress (von Mises stress) were input into the Weka 3.9.2 software. The performance of each model was evaluated using the coefficient of determination (R^2^), mean absolute error (MAE), and root mean squared error (RMSE). For both models, the polynomial kernel (PK), Pearson universal kernel (PUK), and radial basis function kernel (RBF) were used according to Equations (4)–(6).

(4) $$K\left(x,x^{\prime }\right)={\left(x\cdot x^{\prime }+c\right)}^{d}$$

(5) $$K\left(x,x^{\prime }\right)=e^{-\frac{{\left\|x-x^{\prime }\right\|}^{2}}{2\sigma^{2}}}$$

(6) $$K\left(x,x^{\prime }\right)=e^{-\gamma {\left\|x-x^{\prime }\right\|}^{2}}$$

The parameters for each kernel were optimized using the Grid Search method to achieve the best possible performance. To minimize error, the number of iterations in the software was set to 100. The dataset was split such that 70% of the data were used for training the models, while the remaining 30% were allocated for testing.

#### 2.4.4. Impact Test and Finite Element Simulation

The mechanical properties of the films during impact testing were studied by conducting tests on 200 × 200 mm samples. The test was performed using a spherical projectile, whose specifications are given in Table 1.

The projectile was launched from a distance of 50 mm and impacted the center of the samples at a velocity of 2000 mm/s. Based on the initial results, the time for the projectile to collide with the films was recorded as approximately 0.025 s. To analyze and simulate the impact process in more detail, 0.1 s was taken into consideration in the simulation. A friction coefficient of 0.25 was assumed for the interaction between the projectile and the sample surfaces. The simulation was performed using the Dynamic, Explicit step, with a mesh specifically tailored for this condition. Key parameters analyzed in the simulations included the maximum stress (von Mises stress) during impact, strain energy, and plastic dissipation energy. These parameters were examined to gain deep insight into the behavior of the films under impact conditions and to better simulate their performance.

During the impact, a significant amount of the energy applied to the material is stored as strain energy, which is recovered as the material returns to its initial state [24]. This parameter was considered to evaluate the amount of energy stored in the material during elastic deformation and was calculated using Equation (7).

(7) $$E_{strain}=\int_{0}^{\delta_{f}}\sigma \left(\delta \right)d\delta$$

where E_strain_ is the strain energy (J), σ is the stress (Pa), δ is the displacement (m), and $\delta_{f}$ is the final displacement (m) (the point where strain energy is fully stored).

In a simplified case, Equation (8) can be derived and simplified to:

(8) $$E_{strain}=\frac{1}{2}\sigma \delta$$

Plastic dissipation refers to the energy lost due to plastic deformation in the material during the loading and impact process. This parameter is critical for evaluating the material’s behavior under severe loading and irreversible (plastic) changes [25], and it was calculated using Equation (9):

(9) $$E_{plastic}=\int_{\delta_{0}}^{\delta_{p}}\sigma (\delta )d\delta$$

where E_plastic_ is the plastic dissipation energy (J), and $\delta_{0}$ and $\delta_{p}$ represent the initial and final deformations (m), respectively.

#### 2.4.5. Thermal Tests and Finite Element Simulation

- Transient Thermal Analysis

To evaluate the heat transfer in composite films with respect to time, which results in mechanical changes, a transient thermal analysis was performed. This method is highly applicable for simulating time-dependent processes, such as heating or cooling of materials [26]. The time-dependent heat transfer equation used for this analysis is presented in Equation (10).

(10) $$\frac{\partial \varepsilon }{\partial t}\beta +\left(k\nabla T\right)\cdot \nabla =\frac{\partial T}{\partial t}\rho c$$

where ε is the strain, β is the strain-induced heat transfer coefficient (1/K), k is the thermal conductivity (W/m·K), ∇T is the temperature gradient (K/m), ∇ is the thermal field divergence (1/m), T is the temperature (K), t is time (s), ρ is density (kg/m^3^), and c is the specific heat (J/kg·K).

In this analysis, temperature changes and thermal expansion significantly influence stress development. The thermal stress resulting from temperature changes was calculated using Equation (11).

(11) $$\sigma_{thermal}=E\cdot \alpha \cdot ∆T$$

where $\sigma_{thermal}$ is the thermal expansion stress (Pa), E is the modulus of elasticity (Pa), α is the coefficient of thermal expansion (1/K), and ∆T is the temperature change (K).

In thermal simulation, the required parameters of thermal conductivity and specific heat were obtained for each material from reliable scientific sources (Table 2). These values were then calculated for the composite films using the rule of mixtures.

Thermal expansion was determined using the standard formula in Equation (12).

(12) $$\alpha =\frac{∆L}{L_{0}\cdot ∆T}$$

where ∆L is the change in length (m), and L_0_ is the original length (m).

Material properties were derived using experimental data obtained from mechanical tests and subsequently incorporated into the software. The model was designed as a thin sheet with dimensions of 200 × 200 mm. The initial temperature of the model was set to 20 °C, and a constant temperature of 100 °C was applied to the left edge. To ensure stability, the right edge of the model was constrained, with boundary conditions defined as x = y = z = 0. The analysis was configured as a coupled temperature displacement simulation, with the transient analysis option enabled. This approach simultaneously considers heat transfer and mechanical deformation.

The total duration of the process was set to 120 s, with the maximum allowable temperature change per increment restricted to 5 °C to improve simulation accuracy. For meshing, a specialized mesh tailored for coupled temperature displacement analyses was employed to ensure precision. All simulation parameters and conditions were carefully calibrated to examine the thermal behavior of the composite films under the specified conditions.

- Steady-State Thermal Analysis

A steady-state thermal analysis was conducted to assess stable heat transfer within the composite films. In this type of analysis, temperature variations over time are disregarded, and the system is assumed to be in thermal equilibrium [32,33]. The steady-state heat transfer equation (Equation (13)) was used for the calculations.

(13) $$Q=\left(k\nabla T\right)\cdot \nabla$$

where Q is the rate of heat generation (or absorption) per unit volume within the system (W/m^3^).

Input parameters were the same as in the transient analysis. The instantaneous temperature increase up to 100 °C was uniformly applied to the model from the left edge. The analysis type in the Step module was set to heat transfer, with the method specified as steady-state. A specialized mesh designed for heat transfer analyses was employed to ensure accurate thermal calculations.

Following the simulation, Equation (13) was used to calculate the temperature at each node of model NT11. Additionally, the temperature gradient, which illustrates the variations in temperature across the film’s surface, and the heat flux, representing the rate of heat transfer through the material, were determined using Equations (14) and (15), respectively [33,34].

(14) $$\nabla T=(\frac{\partial T}{\partial x},\frac{\partial T}{\partial y},\frac{\partial T}{\partial z})$$

where x, y, and z represent the temperature changes along the x-, y-, and z-axes, respectively.

(15) q=−k·∇T

where q represents the heat flux (W/m^2^), and the negative sign indicates that heat transfer occurs in the direction of decreasing temperature.

## 3. Results and Discussion

### 3.1. Tensile Test Results and Finite Element Simulation

In this study, nine composite films with different compositions and ratios of starch, agar, and hemp fiber powder were fabricated and characterized. The physical and mechanical properties of these films are shown in Table 3. Among all the samples, the pure starch film (S100) exhibited the lowest tensile strength, with a Young’s modulus of 7.68 MPa and elongation at break of 9.90%, confirming its brittle and mechanically weak nature.

**Figure 2:**
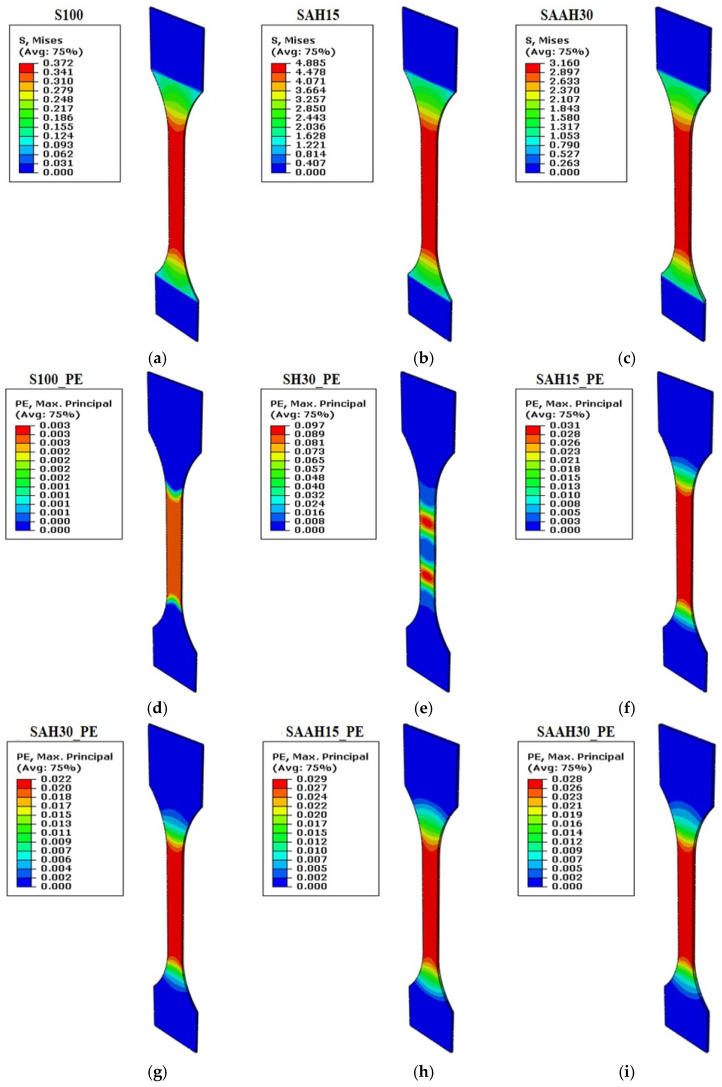
Tensile test simulation for starch/agar composite films reinforced with hemp fibers: (**a**–**c**) maximum stress values; (**d**–**i**) plastic deformation.

Figure 2a shows the distribution of maximum stress during tensile testing for this sample, which was quite homogeneous and concentrated in the central region. The addition of hemp fiber filler to starch (at 15% and 30% concentrations) significantly increased Young’s modulus, which expresses the stiffness of the films. However, the tensile strength decreased when the hemp fiber content increased from 0 to 15%, then increased at the highest content of 30%. Such behavior indicates that, while hemp fiber enhances film stiffness, lower concentrations (up to 15%) may introduce brittleness simultaneously. In lower volume fractions, the fibers may not be uniformly dispersed within the polymer matrix and could result in clustering. These clusters can act as points of stress concentration, hence reducing tensile strength. At higher fiber content, the distribution of fibers within the matrix becomes more uniform, and improved adhesion between the fibers and polymer matrix is achieved. In this case, the fibers act as effective reinforcements, enhancing tensile strength. Pure hemp fibers have been shown in numerous studies to have high elastic modulus and tensile strength, with modulus values ranging from 1.7 to 4.8 GPa and tensile strength values between 23.54 and 930 MPa [35,36,37,38]. These findings demonstrate the important contribution fibers make to improving the mechanical characteristics of biocomposites. The results obtained suggest that the addition of 30% raw hemp fiber had almost no effect on the tensile strength of chitosan-based composites compared to the control film (CH). The highest tensile strength values were observed at 50% hemp fiber content, where the tensile strength of the chitosan layers increased by 34–65% [39]. Luzi et al. (2016) [40] showed that the tensile strength of PLA films reinforced with cellulose nanocrystals extracted from hemp fibers was lower than that of neat PLA, while Young’s modulus and the values of elongation at break were influenced by the fiber content incorporated into the composite.

Adding 50% agar (by weight of starch) to the film formulation significantly improved the mechanical properties of samples SAH0, SAH15, and SAH30 in relation to Young’s modulus, tensile strength, and elongation at break. Specifically, Young’s modulus increased from 48.03 MPa in SH30 to 399.70 MPa in SAH30. The range of stress distribution was also broader for this group compared to S100, suggesting that the addition of agar and fibers reinforced the film structure (Figure 2b). In the SAAH group, increasing the agar content resulted in a reduction in Young’s modulus, tensile strength, and stress distribution range compared to the SAH group, although these values remained higher than those of the SH group (Figure 2c). The presence of agar at specific and limited concentrations increased the homogeneity of the films and improved compatibility between the fiber and matrix. Agar acted like a network-forming polymer, which provided a suitable network in the film and enhanced the bond between polymer chains and fibers. It resulted in the enhancement of Young’s modulus and tensile strength. However, at higher agar concentrations, network saturation and separate phases were formed; these increased the brittleness and reduced the studied parameters. The tensile strength and elongation at break for starch/PVA samples with varying compositions were within the range of 27.41–35.02 MPa and 35.15–41.99%, respectively [3]. Similar results were obtained for agar/PVA films, with tensile strength and elongation at break values ranging from 28.58 to 48.63 MPa and 57.77 to 383.2%, respectively [41].

The second part of Figure 2 presents the plastic strain (PE) distribution during the simulated tensile test for six composite films. In the S100 sample, plastic strain was minimally concentrated in the central region (Figure 2d). This indicates that the sample fractured rapidly before significant plastic deformation could occur. In contrast, the SH30 sample exhibited much higher plastic strain compared to S100, with the strain distribution diagonally concentrated in the central region (Figure 2e). This indicates that the addition of hemp fiber improved the plastic deformation capability of the film, thereby enhancing its flexibility. It is highly attributed to the pretreatment of the hemp fibers due to the removal of lignin and other non-cellulosic parts from the fiber surface, therefore offering improved adhesion between the fibers and the polymer matrix. Bahsaine et al. (2023) [17] reported similar findings for hemp cellulose nanocrystals used as a filler within a chitosan–polyvinyl alcohol (CH/PVA) polymeric matrix. They attributed the development of mechanically robust and flexible bioplastic films to homogeneous dispersion and optimized interfacial contact between the filler and the polymer matrix. Comparable results have also been observed for hemp/PVA films [42].

In SAH30 and SAH15, plastic strain decreased compared to SH30 and was concentrated in the central region of the films (Figure 2f,g). This indicates that the addition of agar to the film formulation reduced plastic strain. In this case, the films stored more energy elastically and exhibited less permanent deformation under load. Increasing the agar content in the SAAH group did not cause significant changes in plastic strain values. This suggests that agar primarily enhances the elastic properties of the material with little effect on plastic strain.

**Figure 3:**
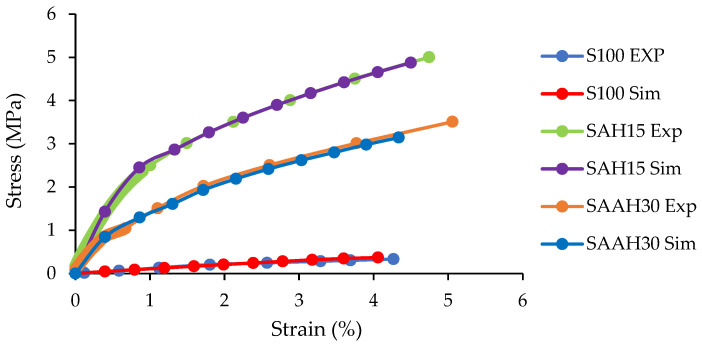
Comparison of experimental and simulated stress–strain curves in the elastic region for three composite films.

Additionally, the increased volume fraction of hemp fiber in this group had minimal impact on plastic strain (Figure 2h,i). This could imply that in the presence of agar, the fibers primarily act as reinforcements, with negligible influence on plastic strain. In other words, the incorporation of hemp fibers alone enhances the plastic deformation capability of the film; this effect is diminished when agar is incorporated. Furthermore, the simulation results for elastic behavior during tensile testing showed close agreement with the experimental data (Figure 3).

This consistency confirms that the simulated model is suitable for analyzing the mechanical behavior of these films. Comparing the stress–strain curves clearly demonstrates that the addition of agar and hemp fibers to the starch films enhanced the slope of the stress–strain curve, thereby improving their tensile strength.

### 3.2. Machine Learning Results for Predicting Tensile Strength Data

**Figure 4:**
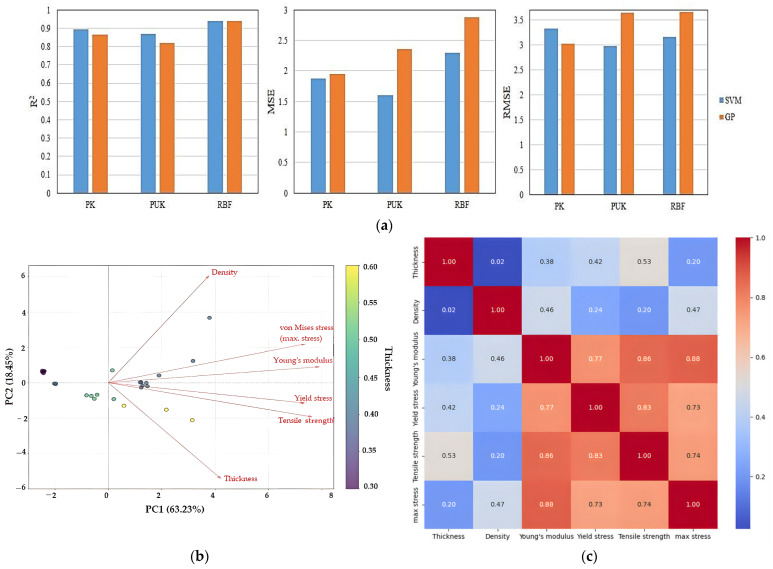
Analysis of starch/agar composite films reinforced with hemp fibers: (**a**) tensile strength prediction using SVM and GP models; (**b**) principal component analysis (PCA); (**c**) correlation matrix of thickness, density, Young’s modulus, yield stress, tensile strength, and maximum stress.

The performance of two machine learning models, SVM and GP, was performed for the prediction of tensile strength in composite films. Overall, these two models have different performance in predicting the tensile strength of the films. As shown in Figure 4a, the SVM model significantly outperformed the GP model. The GP model indeed showed acceptable predictability, while the performance compared to the SVM was somewhat weaker. This could be explained in light of the more complex capacity of high-dimensional regression problems as enabled by the SVM model. In both models, the RBF kernel consistently delivered the best performance compared to the PK and PUK. This superiority was evident across all three evaluation metrics, which include R^2^, MAE, and RMSE.

The RBF kernel’s superior performance in this study can be attributed to its ability to model complex non-linear relationships between input features and tensile strength. Using the Euclidean distance of data points as its basis, the kernel maps data to an infinite-dimensional feature space and then captures complex non-linear relations. On the other side, PK and PUK, dependent upon polynomial relationships, did not develop a good understanding of the non-linear trends in this dataset. GPR and SVR models were used in one study for the examination of the tensile strength of polypropylene composites with wheat straw reinforcement. With 99.13% and 46.28% correlation coefficients, the models demonstrated themselves to be capable of forecasting the values of this characteristic under conditions of a wide range [43]. Artificial neural networks also predicted the mechanical properties of graphene/hemp/epoxy composites with various material ratios and provided the minimum error rate (0.9512) and maximum correlation coefficient (0.9724) [44]. Additionally, with R^2^ = 0.786, the optimal predicted tensile strength performance for resin composites of palm oil reinforced bamboo fibers was provided by the gradient-boosting decision tree model [19]. Applications of the machine learning model demonstrate the potential for efficiently designing and fabricating composites with desired properties in the future without the need for extensive experimental trials.

Figure 4b displays the biplot of the principal component analysis (PCA) performed on the physical and mechanical property data of composite films. The two main components, PC1 and PC2, explained about 63.23% and 18.45% of the total variation of the data, respectively. Consequently, the variation in PC1 can be considered a main factor distinguishing between samples, while the variation explained by PC2 represents a smaller portion of the whole variation. Variables such as Young’s modulus, yield stress, tensile strength, and maximum stress all pointed in a similar direction, indicating their positive relationship among themselves. These variables were predominantly positively correlated with PC1. The vectors representing density and thickness were approximately orthogonal to the other variables, indicating weaker correlation. However, density exhibited a positive correlation with PC2.

The strong positive correlations among Young’s modulus, yield stress, tensile strength, and maximum stress suggest that these properties are highly interrelated within the composite films (Figure 4c). This indicates that changes in composite formulation or processing parameters that serve to increase one of these parameters are likely to improve the others as well. The weaker correlations between thickness and the mechanical properties indicate that simply altering film thickness may not significantly affect the mechanical performance within this set of samples. Density also showed mild correlation with other properties, except for maximum stress. This could indicate that the type of composite material used does not substantially influence density, as it is often expected that more rigid materials have lower densities. Alternatively, the materials in this study may not differ significantly in terms of density. This observation may also imply that density is not a critical parameter in determining the overall mechanical performance, although further data may be required to confirm this conclusion.

### 3.3. Results of Impact Test

#### 3.3.1. Impact Test Results and Finite Element Simulation

**Figure 5:**
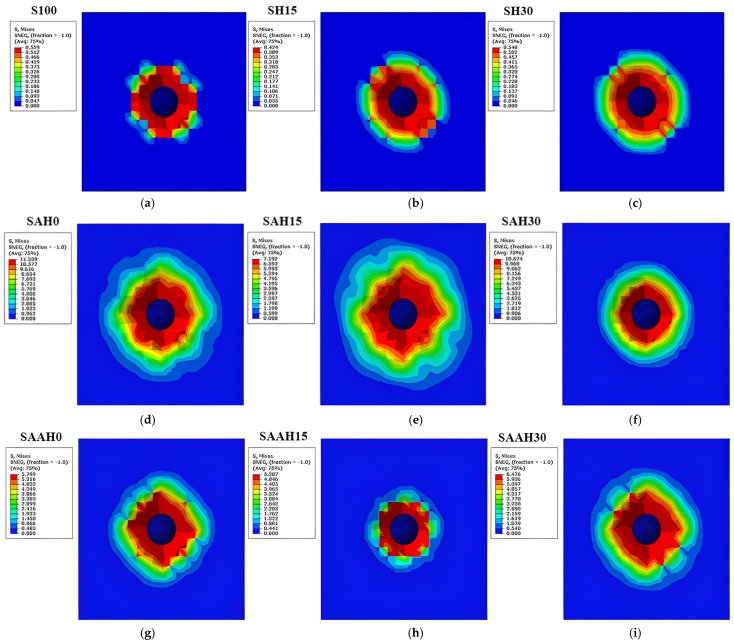
Impact test simulation for starch/agar composite films reinforced with hemp fibers: (**a**–**i**) maximum stress values.

The simulation results of the impact test illustrate the stress distribution patterns in various composite films under impact loading, providing insights into their resistance to impact forces. In the S100 sample, stress was concentrated around the impact area. As shown in Figure 5a, the maximum stress in this sample was lower than in other samples. Due to its weak and brittle structure, this sample exhibited low impact resistance, which was anticipated given its low Young’s modulus and tensile strength. With the addition of hemp fibers in the SH group samples, the stress remained concentrated around the impact area but was more widely distributed compared to the S100 sample. However, in the SH30 sample, stress was not localized at specific points, unlike in SH15, and instead spread continuously across the entire area (Figure 5b,c). In this group, the inclusion of fibers as fillers did not enhance the impact resistance of the films, a finding consistent with the tensile strength results for these samples.

For the SAH0 and SAH15 samples, the stress concentration area was broader compared to the S100 and SH groups, and the stress was distributed throughout the entire film (Figure 5d,e). The addition of agar alone significantly enhanced the impact resistance of the films, aligning with the higher Young’s modulus and tensile strength values observed for these samples. Incorporating 15% fiber into the agar structure created a reinforced configuration that demonstrated both high strength and an excellent ability to distribute stress. In these samples, hemp fiber acted as reinforcing agent, helping to evenly distribute stress within the film structure and preventing stress concentration at the point of impact. In contrast, increasing the fiber content to 30% altered the film structure. In the SAH30 film, hemp fibers no longer functioned effectively as reinforcements, and fiber clustering at the impact zone likely occurred. As a result, stress remained more localized in the impact area and was not evenly distributed throughout the film (Figure 5f). The comparison of stress distribution in SAH15 and SAH30 films shows that the maximum stress level may not be directly correlated with stress distribution, and the material’s behavior can vary depending on the constituents of the composite.

In the SAAH0 and SAAH15 samples, the additional agar content compared to the SAH group resulted in a relative decrease in impact resistance. However, in the SAAH30 sample, the inclusion of hemp fiber partially offset this reduction (Figure 5g–i). Moreover, the improved fiber dispersion within the film structure, facilitated by agar, prevented stress concentration solely at the impact point (unlike in SAH30) and allowed the stress to cover a larger surface area of the film.

These findings highlight the complex behavior of composite materials, where the effects of all contributing factors must be analyzed both independently and interactively.

#### 3.3.2. Strain Energy Variations

**Figure 6:**
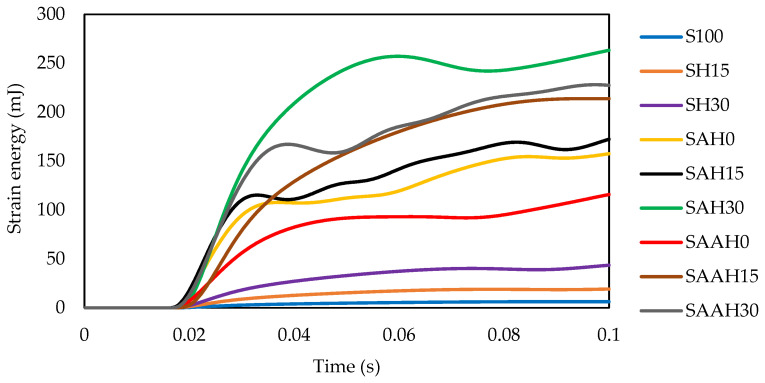
Variations in strain energy obtained from impact tests of starch/agar composite films reinforced with hemp fibers.

The strain energy values of the films as a function of time are plotted in Figure 6. The hemp-fiber-reinforced films demonstrated superior performance regarding strain energy absorption. The SAH30 composition, containing 1.5 g of agar and 30% hemp fiber, exhibited the best performance for energy absorption as a result of elastic deformation. This result is likely attributed to the optimal balance between the strength of hemp fibers and the flexibility of the agar matrix. Samples with low fiber content (e.g., SAAH15 and SAH15) or without fiber (e.g., SAH0 and SAAH0) showed the poorest performance. Films made from pure starch or without agar presented the lowest strain energy values due to the brittle nature of the starch matrix.

Fluctuation in strain energy values was less pronounced in the SAH30 and SAAH15 samples compared to SAAH30, SAH15, and SAH0. This difference can be attributed to the uniform stress distribution and lower stress concentration at the impact site in these samples (Figure 5f,h and Figure 6).

#### 3.3.3. Plastic Dissipation Energy Variations

**Figure 7:**
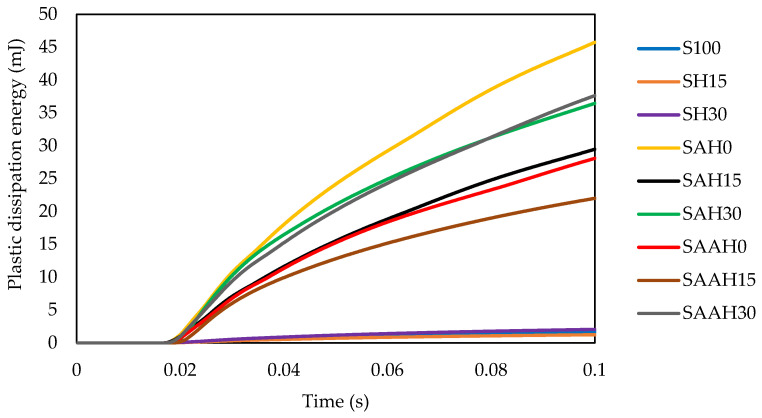
Variations in plastic dissipation energy obtained from impact tests of starch/agar composite films reinforced with hemp fibers.

The plastic dissipation energy values increased non-linearly for composite films subjected to impact, as shown in Figure 7. The most significant increase was observed in the SAH0, SAAH30, and SAH30 samples. Meanwhile, the SAH15, SAAH0, and SAAH15 samples occupied intermediate positions, with values of 29.46, 28.08, and 21.99 mJ, respectively. The presence of agar in the film structure enabled the films to absorb impact energy by plastic deformation. On the other hand, increasing the volume fraction of hemp fibers led to a reduction in plastic dissipation energy, as the matrix became stiffer and less capable of plastic deformation.

Although the pretreatment of hemp fibers with alkali and hydrogen peroxide improved fiber-to-matrix adhesion, the inherent rigidity of hemp fibers led to reduced flexibility and, consequently, plastic dissipation energy. Compositions such as SAH15 and SAAH15, with balanced proportions of agar and hemp fibers, appear to achieve an optimal balance between strength and flexibility, enabling efficient plastic energy absorption.

The minimum plastic dissipation energy was presented for the S100 film due to its brittle structure and lack of reinforcement and the SH15 film with 15% hemp fiber without agar. These samples may be suitable for applications where minimal plastic energy absorption is required.

### 3.4. Results of Thermal Analysis

#### 3.4.1. Steady-State Thermal Analysis

**Figure 8:**
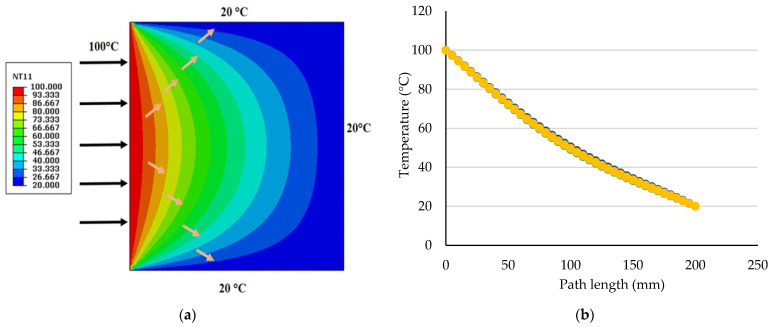
Temperature distribution pattern in steady-state condition for starch/agar composite films reinforced with hemp fibers: (**a**) simulated results; (**b**) numerical values along the elements.

The temperature distribution within the 200 × 200 mm composite films is illustrated in Figure 8a. Heat is transferred from the heated left edge to the center and the rest of the film, consistent with the defined boundary conditions. The temperature distribution in all films is symmetrical and uniform due to the symmetric structure of the films. As demonstrated in Figure 8b, the temperature varies non-linearly across the film elements.

**Figure 9:**
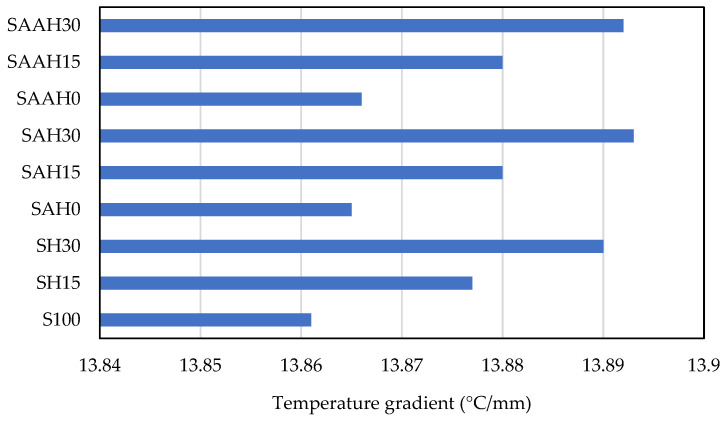
Steady-state thermal analysis of starch/agar composite films reinforced with hemp fibers: temperature gradient.

The temperature gradient results, representing temperature variations across the film thickness, revealed that this parameter depends on the material composition and structure. The temperature gradient for all films ranged from 13.84 to 13.89 °C/mm (Figure 9), indicating relatively uniform temperature variations across the films with no significant differences in temperature distribution among them.

**Figure 10:**
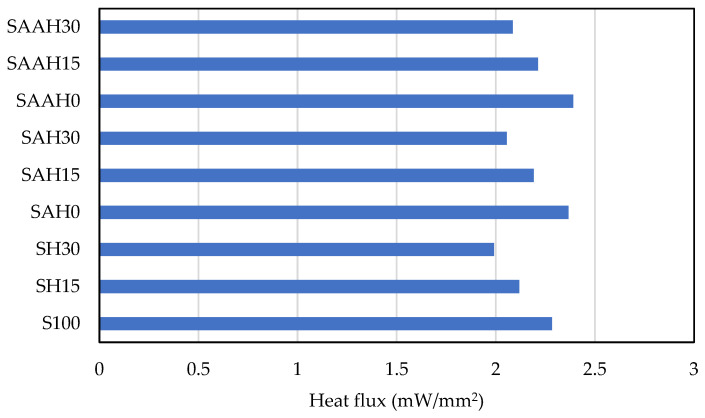
Steady-state thermal analysis of starch/agar composite films reinforced with hemp fibers: heat flux values.

Analysis of the heat flux, which represents the rate of heat transfer through the films, demonstrated that a high volume percentage of hemp fiber (30%) increased the thermal resistance of the films and reduced the heat flux passing through them (Figure 10). These findings demonstrate that optimizing the material composition within the film structure can achieve desirable thermal properties for various applications.

#### 3.4.2. Transient Thermal Analysis

The transient heat transfer simulation results revealed that different composite films exhibit distinct patterns of thermal stress distribution (including tensile and compressive stresses) in response to applied heat. This thermomechanical analysis highlights the apparent changes in all samples at the location of applied thermal stress.

**Figure 11:**
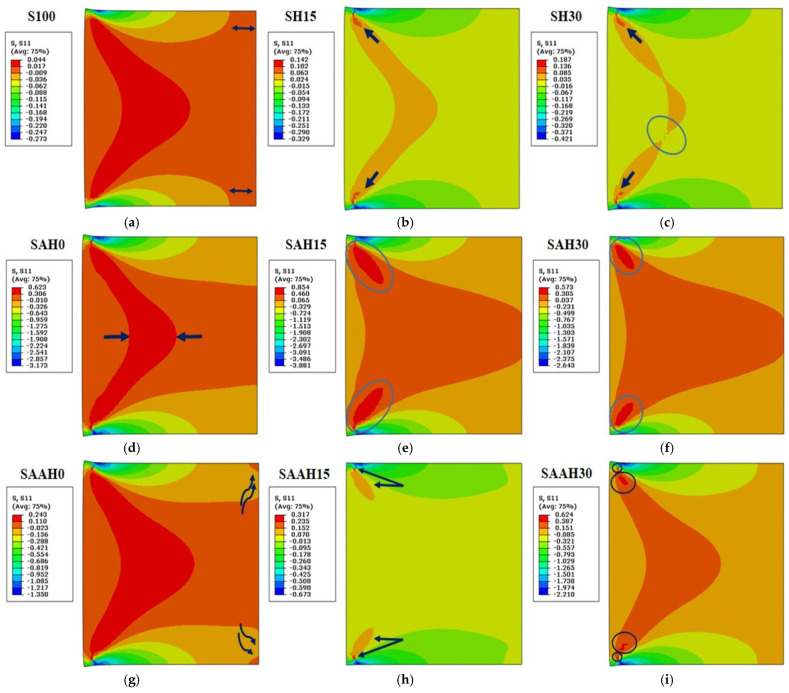
Transient thermal analysis simulation for starch/agar composite films reinforced with hemp fibers: (**a**–**i**) maximum thermal stress values.

In S100, the boundary between tensile and compressive stresses is clearly visible. The central regions, heated edge, and cold edge are subjected to tensile stresses, while the upper and lower parts of the film are symmetrically under compressive stresses. The maximum stress, shaped like a boomerang, is aligned with the direction of heat application and extends toward the center of the sample (Figure 11a). This result indicates that the pure starch structure undergoes less thermal stress compared to reinforced structures. The simulation results for the SH group samples are similar to those of S100, except that the maximum thermal stress has increased in the direction of applied heat, likely due to the addition of hemp fiber. Additionally, the concentration of maximum stress at the heat application area has decreased, and this accumulation is limited to both sides of the heated edge (Figure 11b). From Figure 11c, it can be inferred that increasing the volume percentage of fiber beyond 30% may lead to the degradation of the boomerang-shaped stress region.

In the SAH group samples, the addition of agar to the polymer matrix significantly increased the thermal stress in the films (Figure 11d–f). Tensile stress was dominant in most areas of these samples, indicating effective interaction between the fiber and the matrix. Adding fiber to the agar structure at a moderate level (15%) resulted in a structure capable of withstanding the highest thermal stress (0.854 MPa). The stress distribution pattern in this group is similar to the SH group, except that the intensity of variations has decreased. In SAH0, the concentration of maximum stress in the heat application region resembles a boomerang (thinner than in S100), and as the fiber volume percentage increases, this shape has disintegrated and shifted to both sides of the heated edge. The thermal stability of montmorillonite clay–polyvinyl alcohol biocomposite films was significantly enhanced with the incorporation of rice husk filler up to 6 wt.% [45]. Furthermore, the increased crystallinity of cellulose in hemp fibers further improved the thermal stability of the films [46].

The SAAH group exhibited slightly different results compared to the previous two groups. Although the simulation of the fiber-free sample in this group had similarities with S100, the compressive stress region resulting from applying 100 °C heat had completely reached the cold edge of the sample, and tensile stress was only present at the ends of the edges (Figure 11g). These changes are attributed to the presence of agar in the sample composition as a complementary matrix. The SAAH15 sample, both numerically and in simulation, lies between SH15 and SAH15. Compressive stress was dominant in most areas of this sample. Tensile stress was formed in an elliptical shape in the heat application region and on both sides of the heated edge, and the maximum stress was concentrated in specific and limited points (Figure 11h). This situation indicates the dominance of the agar-starch matrix and the relative influence of the fiber in the thermal testing of this sample. The increase in maximum thermal stress and the growth of tensile stress relative to compressive stress are noticeable as the fiber volume percentage increases from 15% to 30% in this group of films. In SAAH30, the film demonstrates increased resistance to applied stresses, with a maximum stress value approximately equal to that of SAH0 (Figure 11i). The primary difference between these two films lies in the greater resistance of SAH0 at the heat application area, likely due to the presence of hemp fiber. Therefore, in the design and production of composite films, considering thermomechanical aspects, these two samples can be used interchangeably.

## 4. Conclusions

This study developed and characterized biodegradable composite films using starch, agar, and alkaline/peroxide-treated hemp fiber powder. The results showed that the addition of hemp fiber significantly improved the mechanical properties of the starch matrix. Specifically, 30% hemp fiber increased Young’s modulus, while lower concentrations (15%) initially decreased the tensile strength.

The most promising results were observed in the combination of agar with hemp fiber, especially in the SAH group, where the addition of 1.5 g of agar dramatically improved mechanical performance. The SAH30 formulation achieved the highest values for both tensile strength and Young’s modulus. On the contrary, the excess addition of agar in the SAAH group decreased such properties, outlining an optimum in the agar concentration. Machine learning models (SVM and GP) were successfully applied to predict the tensile strength of the samples, with the SVM model using the RBF kernel achieving superior results. The addition of agar also increased impact resistance. Stress distribution and energy absorption were most balanced in the SAH group composition.

Transient and steady-state thermal analyses showed that incorporating hemp fiber increased the thermal resistance of the films. Stress distribution under thermal loading was influenced by material composition, particularly the combination of agar and hemp fiber. Generally, this work underlines the potential of hemp fiber combined with agar in developing enhanced starch-based films, offering promising pathways toward sustainable, high-performance biodegradable materials. The optimization of the balance between agar and hemp fiber content enables the achievement of desirable mechanical and thermal properties. Long-term performance and various field applications of the developed films need further research.

## Figures and Tables

**Table 1:** Physical and mechanical properties of the spherical projectile used in impact tests.

Diameter (mm)	Density (g/cm^3^)	Young’s Modulus (MPa)	Yield Stress (MPa)
20	1.2	2000	70

**Table 2:** Conductivity (W/m·°C), specific heat (J/g·°C), and thermal expansion (1/°C) of composite films at 20 °C and 100 °C [27,28,29,30,31].

	S100	SH15	SH30	SAH0	SAH15	SAH30	SAAH 0	SAAH 15	SAAH 30
Conductiviy (20 °C)	0.05	0.0506	0.0511	0.0530	0.0532	0.0534	0.0538	0.0540	0.0541
Conductivity (100 °C)	0.19	0.175	0.163	0.196	0.180	0.168	0.198	0.182	0.171
Specific heat (20 °C)	1.8	1.73	1.68	1.93	1.85	1.78	1.97	1.88	1.82
Specific heat (100 °C)	2.33	2.24	2.18	2.50	2.40	2.31	2.55	2.44	2.36
Expansion (20 °C)	3 × 10^−5^	3.3 × 10^−5^	3.1 × 10^−5^	4 × 10^−5^	3.7 × 10^−5^	3.3 × 10^−5^	4.1 × 10^−5^	3.8 × 10^−5^	3.6 × 10^−5^
Expansion (100 °C)	6.5 × 10^−5^	6.1 × 10^−5^	5.9 × 10^−5^	6.6 × 10^−5^	6.3 × 10^−5^	6 × 10^−5^	6.7 × 10^−5^	6.3 × 10^−5^	6.1 × 10^−5^

**Table 3:** Physical and mechanical properties of starch/agar composite films reinforced with hemp fibers.

Sample	Thickness (mm)	Density (g/cm^3^)	Young’s Modulus (MPa)	Tensile Strength (MPa)	Elongation at Break (%)
S100	0.30	1.14	7.68	0.511	9.90
SH15	0.35	1.10	28.51	0.407	2.77
SH30	0.40	1.04	48.03	0.556	2.14
SAH0	0.42	0.93	322.11	11.53	22.16
SAH15	0.45	0.95	343.72	7.07	9.60
SAH30	0.45	4.10	399.70	10.86	7.91
SAAH0	0.50	0.89	142.73	5.78	22.30
SAAH15	0.50	4.87	184.82	5.28	14.20
SAAH30	0.60	1.22	234.37	6.46	13.32

## Data Availability

The original contributions presented in the study are included in the article, further inquiries can be directed to the corresponding authors.
